# An Overview of Antimicrobial Properties of Carbon Nanotubes-Based Nanocomposites

**DOI:** 10.34172/apb.2022.049

**Published:** 2021-07-03

**Authors:** Mansab Ali Saleemi, Yeo Lee Kong, Phelim Voon Chen Yong, Eng Hwa Wong

**Affiliations:** ^1^School of Biosciences, Faculty of Health and Medical Sciences, Taylor’s University Lakeside Campus, 47500 Subang Jaya, Selangor Darul Ehsan, Malaysia.; ^2^Department of Engineering and Applied Sciences, American Degree Program, Taylor’s University Lakeside Campus, 47500 Subang Jaya, Selangor Darul Ehsan, Malaysia.; ^3^School of Medicine, Faculty of Health and Medical Sciences, Taylor’s University Lakeside Campus, 47500 Subang Jaya, Selangor Darul Ehsan, Malaysia.

**Keywords:** Carbon nanotubes, Functionalization, Pathogens, Antimicrobial mechanisms, Toxicity

## Abstract

The development of carbon-based nanomaterials has extensively facilitated new discoveries in various fields. Carbon nanotube-based nanocomposites (CNT-based nanocomposites) have lately recognized as promising biomaterials for a wide range of biomedical applications due to their unique electronic, mechanical, and biological properties. Nanocomposite materials such as silver nanoparticles (AgNPs), polymers, biomolecules, enzymes, and peptides have been reported in many studies, possess a broad range of antibacterial activity when incorporated with carbon nanotubes (CNTs). It is crucial to understand the mechanism which governs the antimicrobial activity of these CNT-based nanocomposite materials, including the decoupling individual and synergistic effects on the cells. In this review, the interaction behavior between microorganisms and different types of CNT-based nanocomposites is summarized to understand the respective antimicrobial performance in different conditions. Besides, the current development stage of CNT-based nanocomposite materials, the technical challenges faced, and the exceptional prospect of implementing potential antimicrobial CNT-based nanocomposite materials are also discussed.

## Introduction

Carbon is one of the most readily available elements in nature.^1^ Over the past decades, carbon-based nanomaterials have attracted great attention from researchers and scientists due to their remarkable physicochemical characteristics.^2,3^ There are a wide range of carbon nanostructure (CNS) materials have been discovered for various application, such as carbon nanotubes (CNTs), nano-diamond, fullerenes, carbon nano-onions, nanofibers, and others carbon-based nanomaterials.^3,4^ Among them, CNT is one of the most extensively used materials, especially in biomedical field.^5,6^ CNTs are characterized as hollow and concentric cylindrical structures formed by rolled graphene sheets with a remarkable high aspect ratio.^7,8^ CNTs can be metallic and semi-conductive properties based on the rolling angle.^9^ The classification of CNTs depends on the number of graphene sheets that roll upon their surfaces, such as single-walled carbon nanotubes (SWCNTs) and multi-walled carbon nanotubes (MWCNTs).^10^ The latter comprises multiple single-walled nanotubes that are clustered with each other inside the tubes. Among different types of CNTs, the antimicrobial activity of SWCNTs is higher due to its greater physicochemical properties.^11-14^ Kang et al demonstrated the first report on the antimicrobial activity of purified SWCNTs, that the purified form of SWCNTs and MWCNTs showed significant impact on the integrity of bacterial membranes upon direct contact.^15^ In addition, the morphology and metabolic activities were also compromised.^16^ In their work, the antimicrobial effect of SWCNTs seemed to be stronger than MWCNTs, probably due to their small size which provides a larger surface area to facilitate the membrane perturbation. Besides, the oxidative stress plays an additional role in the CNTs’ antimicrobial mechanisms.^17^ Haung et al investigated the mechanical effects that influenced the antimicrobial properties of CNTs, such as low wear rates, low friction coefficients, favorable tribological characteristics, and high corrosion resistance.^18^ Based on the studies conducted by Chen et al, the SWCNTs played a significant role as “nano-darts” which penetrated bacterial cell walls, reduced membrane potential, released intracellular constituents (DNA and RNA), and ultimately disrupted the bacterial membrane.^19^ More studies have suggested that MWCNTs displayed no mutagenic impact in microbial assays with *S. typhimurium* and *Escherichia coli*.^20^ However, inhibition of microbial growth by CNTs depends on the concentration and treatment time.^21^ On the other hand, the distinguishing characteristic of sp^2^ carbon-based nanomaterials (including CNTs) exhibits exceptional electronic structure that causes semi-conductivity and pseudo metallic conductivity. Vecitis et al investigated this aspect and found that the metallic CNTs were demonstrated higher antimicrobial activity as compared to semi-conducting nanotubes.^22^ Thus, the electronic effects also play an important role in the antimicrobial activity of CNTs. However, the photosensitization process may also activate the CNTs which causes the formation of reactive oxygen species (ROS) in bacterial cells.^23^

Determining the dispersibility of the fibrous colloids is particularly necessary for CNTs. Unmodified CNTs are amphiphobic in nature and insoluble in most of the solvents. Therefore, the dispersion of CNTs may cause aggregation to occur that describes the interfacial surface area with pathogens.^17^ There were a few studies discriminated between aggregated and dispersed CNTs, indicating the diverging of microbial toxicity based on different dispersibility of CNTs.^22^

CNTs have been extensively used in a wide range of medical and pharmaceutical fields.^24-27^ A large variety of nanocomposites, such as silver, enzymes, antimicrobial peptides (AMPs), and polymers are adsorbed on the surface of CNTs to increase the antimicrobial activity of nanotubes.^26,28,29^ Up to date, a promising new method has been developed to resolve the antimicrobial resistance by combining bioactive molecules or antimicrobial drugs with CNTs and then developing new antimicrobial therapy options.^5,28,30^ In addition, toxicological assessments of CNTs should be taken into consideration especially in the presence of catalytically active iron and other possible byproducts that were embedded in the nanotubes.^31^

The covalently functional groups or molecules which are adsorbed on the surface of nanotubes significantly alter the microbial reactions,^32^ which will be further discussed in this review. Besides, the previous studies on the interaction behavior of CNT-based-nanocomposites with microorganism, antimicrobial activity, the toxicity/biosafety profile of modified CNTs, the current research trend, and the development potential of CNTs will also be reviewed in this work.^33,34^

The main challenge of using CNT-based-nanocomposites in antimicrobial research is that the raw CNTs are insoluble in any solvent due to the strong van der Waals interactions among nanotubes. In conjunction with their hydrophobic characteristic, CNTs do not disperse in solution but to form bundles or aggregates, as mentioned earlier. Undoubtedly, such hydrophobic characteristic and the intermolecular attractions between tubes should initially be overcome by any means.^35^ The lack of solubility or dispersibility of CNTs, especially in water, will vastly restrict their use in biological and biomedical applications. On the other hand, it precludes the notion of derivatization of CNTs, which provides the opportunity to conjugate various bioactive molecules, such as therapeutic drugs, targeting ligands and proteins.^36^ Hence, some promising and devising strategies are required to prevail over these limitations to pave the way of utilizing these organic materials as drug delivery systems.

## Antimicrobial performance of pristine CNTs

CNTs are one of the competitive nanomaterials which have been extensively used in the development of antimicrobial surfaces. The antimicrobial activity of CNTs depends on various factors including their composition and surfaces such as length, size, number of graphene layers, and physical disposition (dispersion or aggregation).^17,19,37^ Table 1 lists several studies on the antimicrobial properties of SWCNTs and MWCNTs against different microbial pathogenic strains.

**Table 1:** The antimicrobial performance of pristine carbon nanotubes in different studies

**Types of CNTs**	**Synthesis method**	**Concentration**	**Species**	**Main findings**	**References**
SWNTs	CO disproportionation	5 µg/mL	*E. coli*	Releasing intracellular content due to irrecoverable outer membrane damage.	^15^
SWNTs	CO disproportionation	5 µg/mL	*E. coli*	Microbial cells lost their cellular integrity.	^16^
MWNTs	CVD method	5 µg/mL	*E. coli*	Many of the bacterial cells remain intact and preserve their outer membrane.	^17^
SWNTs and MWNTs	CVD method	20 µg/mL, 50 µg/mL, 100 µg/mL	*L. acidophilus*, *E. coli*, *B. adolescentis, E. faecalis*, and *S. aureus*	The antimicrobial mechanism is associated with length-dependent wrapping and diameter-dependent piercing upon microbial cell membrane damage and the release of intracellular contents.	^19^
MWNTs	Nanocycle productions	1.5 mg/L^-1^ – 100 mg/L^-1^	*E. coli*	The MIC values of MWNTs were high, indicating low microbial toxicity.	^38^
MWNTs	-	-	*E. coli, B. subtilis,* and *P. aeruginosa*	The viability results demonstrated that the toxicity of MWNTs (2-log cell density reduction) against selected pathogens.	^39^
DWNTs and MWNTs	NE scientific productions	20 µg/mL – 100 µg/mL	*Staphylococcus aureus*, *P. aeruginosa*, *K. pneumoniae*, and *C. albicans*	MWNTs demonstrated higher antimicrobial activity than DWNTs against selected pathogens.	^21^
MWNTs	Nanotech Labs productions	20 mg/20 mL	*P. fluorescens*	The percentage of inactivated bacteria by MWNTs was recorded at 44%. It was observed that MWNTs showed a significant effect on the inhibition of microbial adhesion due to the electrochemical potential.	^40^
SWNTs	-	5 µg/mL	*Escherichia coli*, and *Bacillus subtilis*	No obvious physical destruction was observed below 10 nN of applied force.	^41^
SWNT, DWNT, and MWNT	Electric arc discharge, and CCVD	100 µg/mL	*Staphylococcus aureus, P. aeruginosa,* and *C. albicans*	Microbial death induced by the aggregation of CNTs that were trapped on the microbial cell surface.	^42^
SWNTs, and MWNTs	-	0.2 mg/mL	*E. coli*	Laser-activated CNTs had the potential to control the growth of bacteria.	^43^

Up to date, various mechanisms have been suggested to quantify the CNTs toxicity and their biosafety. In 2007, Kang et al reported for the first time that single-walled CNTs were showed strong antimicrobial activity, which caused cell membrane destruction via direct contact and thus, reducing the cell viability by 80%.^15^ In 2008, another study on the gene expression analysis showed that the impairment of cell membrane is the main mechanism of CNT-biocidal. The authors found that the pathogens exposed to CNTs are exhibited oxidative stress, accompanied by the destruction of cell membranes and release of intracellular contents.^17^ Nagai and Toyokuni also reported the occurrence of the impairment of cell membrane by direct piercing of the pathogen surface.^44^ On the other hand, it has been reported that the length of CNTs plays a crucial role in their interactions with the cellular membrane, where longer tubes demonstrate lower toxicity to the pathogen.^4,16^ In addition, Aslan et al observed that the toxicity of shorter SWCNTs is greater due to the higher density of open tube ends.^26^ This observation has been supported by another study,^45^ where nanotubes with smaller diameter tend to cause the destruction of underlined cell membrane via cellular surface interaction. Besides, the microbial death can be induced by the aggregation of CNTs that are trapped on the microbial cell surface.^41,42^ While on the other hand, Arias et al found that CNTs with a larger diameter of 15-30 nm were interacted with pathogens mainly via their sidewalls.^46^

Similarly, many studies have shown that SWCNTs are highly toxicity to the pathogen than MWCNTs and convincingly causing the destruction of the cell membrane of pathogen.^17,19^ In the study carried out by Kang et al, the majority of *E. coli* bacterial cells were flattened after incubated with SWCNTs for one hour but remained intact when incubated with MWCNTs.^17^ In addition, they also observed that *E. coli* exhibited higher level of stress-related genes in the presence of SWCNTs, as compared to MWCNTs.

In contrary to the studies mentioned above, Young et al proposed that the toxicity of MWCNTs is greater for bacteria as compared to SWCNTs.^47^ Saleemi et al have recently studied on the antimicrobial effects of CNTs (DWCNTs and MWCNTs) against different microbial strains, such as *Staphylococcus aureus*, *Pseudomonas aeruginosa*, *Klebsiella pneumoniae*, and *Candida albicans.*^21^ It was reported in their study that non-covalently dispersed MWCNTs exhibited higher antimicrobial activity than DWCNTs.

Despite the inconsistent reports, CNTs still remain competitive among other nanomaterials in fighting against a broad range of microorganisms in view of the fact that their antimicrobial activity has been traced and confirmed on various microbial strains, including *S. aureus*,* E. coli*,* Enterococcus faecalis*,* Lactobacillus acidophilus*, and *Bifidobacterium adolescentis*.^15,17,19^ However, their broad-spectrum antimicrobial properties against various types of pathogens need to be further investigated.

## Antimicrobial properties of functionalized SWCNTs-based nanocomposites

The significant antimicrobial properties of SWCNTs should be highlighted as an effective antimicrobial agent to inhibit the growth of microorganisms on different biomedical surfaces. Their interaction behavior with different types of microorganisms should be further addressed. Therefore, this section will focus on the effect of functionalized SWCNTs with different nanocomposites to increase their antimicrobial ability towards various microbial strains, as summarized in Table 2.

**Table 2:** Overview on the antimicrobial activity of functionalized SWCNTs-based nanocomposites in different studies

**Material blend**	**Concentration**	**Species**	**Main findings**	**References**
f-SWNTs with functional groups (-OH, -COOH, -NH_2_)	50-200 µg/mL	*S. aureus, B. Subtilis,* and *S. typhimurium*	SWNTs functionalized with -OH and -COOH functional group showed more microbial inhibition rate (7-log reduction) against selected pathogens, while SWNTs with -NH_2_ displayed antimicrobial activity only at high concentrations.	^46^
Silver-SWNTs functionalized with peptides (TP226, TP359, TP557)	5 µg/mL	*S. aureus*	The viability of bacteria increased by 4-log in non-treated skin model, whereas treated skin with functionalized silver-SWNTs showed antimicrobial activity only 1-log reduction.	^28^
Functionalized SWNTs with DNA and lysozyme (LSZ)	~25 mg/L	*S. aureus,* and * M. lysodeikticus*	Layer by layer coating of DNA- and LSZ-SWNTs displayed high antimicrobial activity (with 84% microbial reduction).	^29^
SWNTs incorporated inside poly(lactic-co-glycolic acid)	< 2% by weight	*E. coli*, and *S. epidermidis*	The metabolic activity of bacteria was considerably decreased (98%) with SWNTs-PLGA, while 15-20% reduction rate observed with pure PLGA.	^26^
SWNTs-polyvinyl-*N*-carbazole (PVK) nanocomposite	3 wt.%	*E. coli,* and *B. subtilis*	SWNTs-PVK nanocomposite induced a higher rate of bacterial inactivation (90% for *B. subtilis* and 94% for *E. coli*) in the planktonic cells and showed a significant reduction of biofilm formation.	^48^
SWNTs assembled with poly(L-glutamic acid) (PGA) and poly(L-lysin)(PLL) (layer-by-layer)	< 2% by weight	*E. coli,* and *S. epidermidis*	SWNTs/PGA/PLL showed a higher rate of antimicrobial activity (90%) against selected pathogens than non-treated samples of PGA/PLL (with 20% reduction rate).	^26^
Oxidized SWNTs with poly(vinyl alcohol) (PVOH) nanocomposite	0-10% (w/w)	*Pseudomonas aeruginosa*	The viability of cell deposited on the surface of O-SWNTs-PVOH gradually decreased with increasing in nanotubes loading.	^49^
SWNTs/porphyrin composite	0.04 mg/mL	*S. aureus*	In the presence of visible light, SWNTs/porphyrin induced damage to the cell membrane.	^50^
Functionalized-SWNTs/ poly(ethylene glycol) (PEG) and poly(ε caprolactone) composites	0.5-1.0 wt.%	*P. aeruginosa,* and *S. aureus*	The proliferation of tested bacteria inhibited by f-SWNT/copolymer complex to a lower extent as compared to pure polymer complex.	^51^
SWNTs bound with polyamide membranes	0.1-0.2 mg/mL	*E. coli*	The complex of nanocomposite inactivated the microbial cells by 60% after 1 h of contact time.	^50^

### Functionalized with carboxyl, hydroxyl and amine groups

As mentioned earlier, there are different functionalization methods for CNTs, such as covalent and non-covalent methods. Notably, CNTs can be functionalized/modified with acid/carboxyl moieties for the formation of CNTs-bacterial aggregates to increase their interaction with pathogens.^46^ Previous studies reported that the functionalization of CNTs facilitated their binding with microbial cells.^24,52^ In their work, the effects of various surface functional groups of SWCNTs were studied, including -NH_2_, -COOH, and -OH on their microbial inhibitory effects against *S. aureus*, *B. subtilis*, and *Salmonella typhimurium*. They found that functionalized SWCNTs with cationic – NH_2_ group inhibited bacterial growth only at high concentrations, while the SWCNTs with anionic – COOH and neutral – OH groups demonstrated strong inhibitory effects (7-log reduction) against selected pathogens. The strong inhibitory effects mean that some cells or all cells in the cell population were inactivated after treatment with SWCNTs-COOH and SWCNTs-OH. These surface groups such as – COOH and – OH were derived directly from the surface of SWCNTs, whereas – NH_2_ group was modified with a long chain CH_3_ (CH_2_)_16_CH_2_-NH_2_. They suggested that direct contact with the SWCNTs is the likely mechanism causing bacterial cell death, the long carbon chain may affect the interactions of SWCNTs – NH_2_ group with microbial cells in such a way that cylindrical shape of SWCNTs may not be in close direct contact with microbial cell walls that probably account for the reduced inhibitory effects of SWCNTs – NH_2_. In addition, functionalized SWCNTs tend to promote bacterial interaction with nanotubes irrespective of the surface functional group and their inhibitory effects are presented in a selective manner.^46^

### SWCNTs coated with silver nanoparticles

Many studies have demonstrated the antimicrobial activity of silver nanoparticles (AgNPs) and other metal oxides together with their inhibitory effects on the infections.^24,53^ Chaudhari et al observed that the antimicrobial properties of silver coated SWCNTs can be modified with AMPs against *S. aureus* applied in a skin model.^28^ In their study, the proliferation of bacteria was considerably inhibited by 10^5^ CFU/g of silver coated functionalized CNT after skin treatment.^28^ In general, AgNPs have the tendency to bind and penetrate the bacterial cell membrane and therefore causing cell death by altering the permeability of membrane. Besides, the ROS may also be produced in the process.^54^ Moreover, it has been proven that AMPs show antimicrobial effect on various fungi, bacteria, and viruses.^53^ Hence, the synergistic effects of AgNPs with AMPs increase the toxicity of nanotubes and the findings can be useful for the development of novel antimicrobial therapies.^28^ Chaudhary et al attached SWCNTs to AgNPs and bio-conjugated this approach to AMPs TP359 to evaluate the antimicrobial activity of SWCNTs-adsorbed AgNPs against *S. aureus*, *Streptococcus pyogenes*, *Salmonella enterica* serovar Typhimurium, and *E. coli*.^55^ They found that the conjugation showed a strong synergistic antibacterial effect of TP359 with SWCNTs-adsorbed AgNPs. Another study conducted by Kumar et al reported the antibacterial potency of decorated SWCNTs with AgNPs in cotton fabrics against *S. aureus* and *E. coli*.^56^ They observed that the fabrics coated with SWCNTs-AgNPs showed excellent antibacterial properties against selected pathogens. In addition, AgNPs on silica-coated SWCNTs substrate showed significantly inactivated the bacterial growth (*E. coli*) as compared to AgNPs on plasma-treated SWCNTs substrates that lose their hydrophilicity during AgNPs deposition.^57^ In another study, silver-based biohybrids demonstrated antioxidant and antimicrobial properties against *S. aureus*, *E. coli*, and *E. faecalis*.^58^ The silver-based biohybrids consisting of phytonanosilver, CNTs, and cholesterol-containing liposomes showed higher reduction rates of microbial growth and antioxidant activity.

However, Chang et al used a facile and simple one-step approach for the synthesis of CNTs and graphene oxide with AgNPs against *E. coli* and *S. aureus*.^59^ They observed that the synthesized nanomaterials showed antibacterial activity, but the graphene oxide AgNPs exhibited highest disinfection property. The lipid peroxidation assay and antioxidant enzyme activities proved that the nanomaterials were able to induce O_2_ - oxidative stress on bacteria, thus affected the cell membrane integrity and ultimately caused cell death. Another study showed the antimicrobial properties of SWCNTs coated with Ag-doped TiO_2_ nanoparticles against *E. coli* and *S. aureus*.^60^ The results demonstrated that synthesized nanocomposites have a strong antibacterial activity against both types of bacteria, while *S. aureus* appeared to be less susceptible to the nanocomposite samples than *E. coli* under illumination by UV light. Park et al synthesized pegylated single walled carbon nanotubes (pSWCNTs) coated AgNPs with enhanced antibacterial properties and evaluated their effects on foodborne pathogenic bacteria.^61^ They found a significant reduction in proteins associated with bacterial biofilm formation, quorum sensing and maintenance of cellular architecture, and cell motility in surviving foodborne pathogen. Moreover, Singh et al prepared a hybrid SWCNTs/Ag/PPy based nanocomposite by using a facile and cost-effective one-pot synthesis technique.^62^ The prepared nanocomposites have the ability to inhibit the growth of selected bacterial strains such as *S. aureus*, *P. aeruginosa*, *E. coli* and *B. cereus* completely within 24 h. Zhu et al proposed a new antimicrobial nanoplatform of mesoporous silica-coated and AgNPs-coated SWCNTs developed by a N-[3-(trimethoxysilyl)propyl]ethylene diamine (TSD)-mediated approach (SWCNTs@mSiO_2_-TSD@Ag).^63^ In this system, they compared commercial AgNPs and TSD modified mesoporous coated SWCNTs and found that the nanosystem of SWCNTs@mSiO_2_-TSD@Ag showed a strong antimicrobial activity against multi-drug resistant bacterial strains by damaging the cell membrane of bacteria and eventually a quick release of Ag ions. Yun et al revealed the antibacterial activity of CNTs-Ag and GO-Ag against both gram-positive and gram-negative pathogens.^64^ They observed that antimicrobial activity of CNTs-Ag was higher as compared to GO-Ag nanocomposites that may be due to the excellent dispersion of AgNPs into the CNTs. Another study showed that carbon-Ag nanocomplex prevented the microbial growth against methicillin-resistant *S. aureus*, *K. pneumoniae*, *Acinetobacter baumannii*, and *Burkholderia cepacian*.^65^ These nanocomposites can also be used to prevent the proliferation of bio-defense pathogen such as *Yersinia pestis*.

### Immobilization of enzyme with SWCNTs

CNTs can also be modified with natural enzymes, such as lysozyme (LSZ) to enhance their toxicity towards different bacterial species including *S. aureus*, and *Micrococcus lysodeikticus* as investigated.^29^ The antimicrobial activity of LSZ and its mechanisms comprising of cell wall lysis via hydrolyzing the beta 1, 4 linkages between *N*-acetylglucosamine and *N*-acetylmuramic acid on peptidoglycan have been also previously described.^24,66^

### SWCNTs associated with polymers

Biomaterials that could inactivate the microbial cells are needed to reduce the infections associated with medical devices. The chemical modifications of CNTs improve their dissolution properties and chemical compatibility, while functionalization with polymer enhances the dispersibility and solubility of CNTs as well as increases the interfacial interaction to polymeric matrices in their composites. For instance, SWCNTs in the form of deposited aggregates and membrane coatings have been demonstrated to be highly toxic to the microbial cells. Their ease of modification and chemical stability render SWCNTs attractive nanomaterials to the antimicrobial biomaterials. Moreover, SWCNTs in their pure form are expensive and only provide a limited range of material properties, thus they are unlikely to develop as ideal antimicrobial materials. However, SWCNTs modified with polymers could enhance the antimicrobial property and also provide a wide range of mechanical, degradation, and structural properties.

Aslan et al made SWCNTs nanocomposite with poly (lactic-co-glycolic acid) (PLGA) matrix and investigated their antimicrobial activity against *Staphylococcus epidermidis* and *E. coli*.^26^ They reported that SWCNTs-PLGA had reduced the bacterial viability with a 98% cell reduction as compared to pure PLGA. The metabolic activity of the bacteria was significantly reduced as reported.^26^ Moreover, the SWCNTs association with polyvinyl-N-carbazole caused a higher inactivation rate of planktonic cells (90% for *B. subtilis* and 94% for *E.coli*) and reduced their biofilm formation.^48^ Similarly, nanocomposite prepared by SWCNTs associated with poly(L-glutamic acid) and poly(L-lysine) resulted in high inactivation rate of* E. coli* and *S. epidermidis* up to 90%.^67^ Goodwin et al synthesized a nanocomposite namely SWCNTs-poly(vinyl alcohol) and investigated its antimicrobial activity against *P. aeruginosa*.^49^ The bacterial viability gradually reduced with increasing concentrations of SWCNTs.^49^ Sah et al reported that the formation of SWCNTs nanocomposite with photosensitive molecules (porphyrin) exhibited a sufficient antimicrobial effect against *S. aureus*.^50^ The bacterial cells were treated with porphyrin appended SWCNTs in the presence of visible light using tungsten-halogen lamp. They observed that it formed the short lived first excited state (1PS) after absorption of light by porphyrin and the first excited singlet state encounters the intersystem crossing, resulting in a long-lived excited triple state which is primarily responsible for various chemical reactions. The photochemical reaction initiated by the transfer of electrons from the triple excited state (3PS) to the SWCNTs that inhibits the electron-hole recombination and ultimately transfer the electron to the ambient molecular oxygen to form ROS. The formation of ROS causes destruction of bacterial cell wall that leads to the bacterial cell death.^50^ Furthermore, SWCNTs which were covalently bound with polyamide membranes had successfully inactivated 66% of bacteria and caused a delay in membrane biofouling.^68^ In contrast, previous studies reported that non-ionic surfactant and hydrophilic polymers were suitable materials to adhere the CNTs surface which to be applied in various biomedical applications. For instance, poly(ethylene glycol) (PEG) is the most effective coating agents due to its high hydrophilicity. Cajero-Zul et al used a linear and branched PEGs attached to the surface of CNTs were assessed as effective nanosystems to be used as a new medium.^51^ They observed that bacteria exposed to SWCNTs-copolymer of star-shaped poly (ethylene glycol) (PEG) and poly(ε caprolactone) (PCL) did not demonstrate antimicrobial activity, but the thermal and mechanical properties of nanocomposites were better than the ones of their polymeric matrix. The star-shaped PCL-PEG copolymer structure was assessed by using different techniques, such as FTIR, GPC, ^1^H-NMR, and ^13^C-NMR spectroscopies. Moreover, the molecular structure of star-shaped copolymer enables the poly(ethylene glycol) (PEG) chains be exposed to the bacterial action and prevented their growth.^51^ In view of that, certain SWCNT-nanocomposites show significant antimicrobial activity, while some others display a combination of antimicrobial properties.

## Antimicrobial properties of functionalized MWCNTs nanocomposites

MWCNTs have been widely studied and used in many sectors due to their unique physicochemical properties and antimicrobial potential. MWCNTs functionalized with various materials are extensively studied and implemented to produce effective antimicrobial surfaces.^69,70^ Table 3 summarized the previous studies which were conducted with respect to MWCNTs biocidal effects and their interaction with a broad range of microorganisms.

**Table 3:** Overview on the antimicrobial activity of functionalized MWCNTs-based nanocomposites in different studies

**Material blend**	**Concentration**	**Species**	**Main findings**	**References**
	50-200 µg/mL	*S. aureus, B. subtilis, and S. typhimurium*	MWNTs functionalized with -OH and -COOH functional group did not significantly induce antimicrobial activity on selected pathogens.	^46^
	25 µg/mL	*E. coli, B. subtilis,* and *S. aureus*	MWNTs-COOH inactivated the bacterial cells by 30% for *B. subtilis*, 40% for *E. coli*, and 50% for *S. aureus*, respectively.	^71^
	20 μg/mL	*S. aureus, E. coli,* and * P. aeruginosa*	MWNTs-COOH inactivated the bacterial cells by 26.9% for *P. aeruginosa*, 34.1% for *E. coli*, and 22.8% for *S. aureus*, respectively.	^72^
f-MWNTs with functional groups (-OH, -COOH, -NH_2_)	20 mg/20 mL	*S. aureus, E. coli,* and *P. aeruginosa*	MWNTs-COOH inactivated the bacterial cells by 26.8 ± 1.1 for *P. aeruginosa*, 20 ± 0.8 for *E. coli*, and 14.7 ± 0.5 for *S. aureus*, respectively.	^73^
	20 µg/mL, 50 µg/mL, 100 µg/mL	*E. coli*, *S. aureus*, *E. faecalis*, *L. acidophilus*, and *B. adolescentis*	MWNTs-COOH and MWNTs-OH induced dose-dependent microbial inhibition against selected pathogens.	^21^
	1000 µg/mL	*V. parahaemolyticus*	Antimicrobial activity of functionalized-MWNTs was time-dependent. Functionalized nanotubes that did not pierce into the cell membrane, rather wrapped around the surface of the pathogen.	^74^
	0–100 mg/mL	Group A* Streptococcus*	Carboxylated-MWNTs functionalized with antibodies may have the potential to mitigate the bacterial infections of soft tissue.	^75^
	20 µg/mL – 100 µg/mL	*Staphylococcus aureus*, *P. aeruginosa*, *K. pneumoniae*, and *C. albicans*	Microbial growth was inhibited by non-covalently dispersed CNTs and relied heavily on the treatment time and concentration. MWNTs demonstrated higher antimicrobial effect on selected pathogens.	^21^
Surfactant- functionalized MWNTs with sodium dodecylbenzene sulfonate (SDBS), sodium cholate (SC), sodium dodecyl sulfate (SDS), triton X-100 (TX-100), dodecyltrimethylammonium bromide (DTAB), cetyltrimethylammonium bromide (CTAB), and polyvinylpyrrolidone (PVP)	1.0, 0.5, 0.25 and 0.125 mg/mL	*S. mutans*	Functionalized-MWNTs caused cell membrane rupture via direct contact.	^76^
	0.1, 0.5, 1 mg/mL	*E. coli*	Functionalized-MWNTs penetrated the bacterial cell membrane due to electrostatic forces between bacterial membrane and surfactant.	^77^
AgNPs-coated MWNTs	2-30 wt%	*E. coli*	The cell membrane of bacteria damaged via direct contact.	^78^
f-MWNTs with lysine	0.01875 to 0.6 mg/mL	*S. aureus, E. coli, S. agalactiae, S. typhimurium, S. dysgalactiae,* and *K. pneumoniae*	Electrostatic adsorption presented between the bacterial membrane and positive charges lysine groups on MWNTs.	^79^
MWNTs functionalized with amphiphilic dendrimer poly(propyleneimine)	25 µg/mL	*E. coli, B. subtilis,* and *S. aureus*	MWNTs-nanocomposite inactivated the bacterial cells by 96.5% for *S. aureus*, 96.6% for *B. subtilis*, and 87% for *E. coli*, respectively.	^71^
MWNTs functionalized with aromatic dendrimer polyamide	20 μg/mL	*S. aureus, E. coli,* and *P. aeruginosa*	MWNTs-nanocomposite inactivated the bacterial cells by 35.5% for *S. aureus*, 65.2% for *P. aeruginosa*, and 72.6% for *E. coli*, respectively.	^72^
Poly(amidoamine)-grafted MWNTs	20 mg/20 mL	*S. aureus, E. coli,* and *P. aeruginosa*	MWNTs-nanocomposite complex inactivated the bacterial cells by 60 ± 1.8% for *P. aeruginosa*, 34.1 ± 1.2% for *E. coli*, and 22.8 ± 0.9% for *S. aureus*, respectively.	^73^
Oxidized MWNTs/poly(vinyl alcohol) nanocomposite	0-10% (w/w)	*P. aeruginosa*	MWNTs-poly(vinyl alcohol) was able to reduce the viability of bacteria with increasing concentrations of nanotubes.	^49^
MWNTs-chitosan hydrogels	25, 50, 100 mg/40 mL	*S. aureus, E. coli,* and *C. tropicalis*	MWNTs-chitosan hydrogels exhibited higher antimicrobial activity against *S. aureus* and *C. tropicalis* than *E. coli.*	^80^
	0.01%, 0.1% and 0.2% (w/w)	*E. coli, S. pneumoniae, S. racemosum, C. albicans, P. aeruginosa, E. coli, G. candidum,* and *A. fumigatus*	MWNTs nanocomposite showed strong microbial inhibition rate against Gram-positive bacteria than Gram-negative bacteria.	^81^

### MWCNTs attached with functional groups

The attachment of functional groups to the surface of CNTs is to prevent desorption processes and unwanted absorption of the molecules from the biological medium.^82-86^ The efficacy of microbial growth inhibition depends on the adsorption rate of various functional groups on the surface of CNTs. Pasquini et al assessed the association between functional group attachment to the surface of CNTs and microbial cytotoxicity.^32^ They further corelated the toxicity with physicochemical properties and functionalized SWCNTs agglomeration state, and reported that no direct correlation was identified between the bacterial cytotoxicity and thermal, physicochemical, and structural properties of f-SWCNTs. The aggregation of nanoparticle was superior to the individual chemical and physical properties of functional groups when evaluating the f-SWCNTs cytotoxicity.^32^ Moreover, MWCNTs functionalized with surface functional group (-COOH) have significantly reduced the viability of bacteria by 30% for *B. subtilis*, 27% for *P. aeruginosa*, 20~40% for *E. coli*, and 15~50% for *S. aureus*.^71-73^ Chen et al showed that MWCNTs with functional groups (-OH, -COOH) demonstrated a significant dose-dependent antimicrobial effect on pathogens, such as *E. coli*, *S. aureus*, *E. faecalis*, *L. acidophilus*, and *B. adolescentis*.^19^ Ding et al were also observed the same effect on *Vibrio parahaemolyticus*.^87^ Arias et al reported that MWCNTs functionalized with -COOH, -NH_2_, and -OH did not induce significant antimicrobial activity as compared to SWCNTs.^46^ The antimicrobial activity of non-covalently dispersed CNTs (DWCNTs and MWCNTs) against *S. aureus*, *K. pneumoniae*, *P. aeruginosa*, and *C. albicans* has been extensively studied.^21^ In their findings, the microbial growth which was prevented by non-covalently dispersed CNTs relied heavily on the treatment time and concentration. The functionalized MWCNTs with a compound named ethanolamine, were showed suppression of the microorganisms growth as compared to pristine MWCNTs.^88^ Another study showed that MWCNTs modified with oxygen groups could increase the antimicrobial properties.^89^

### MWCNTs coated with silver nanoparticles

Like SWCNTs, silver-coated-MWCNTs displayed remarkable antimicrobial performance. The data showed that silver/MWCNTs complex inhibited the bacterial growth by 93.7~99% for *S. epidermidis* and *E. coli*, 56.7% for *S. aureus*, 100% for *Sphingomonas spp.* and *Methylobacterium spp.* and 69.7% for *P. aeruginosa*.^73,90,91^ The amphiphilic dendrimers poly(propyleneimine) formed a complex with silver-coated-MWCNTs that inactivated the bacteria by percentage of > 90% for *S. aureus*, *B. subtilis*, and *E. coli*.^71^ Similarly, polymer colloids immobilization with silver/MWCNTs complex exhibited strong antimicrobial effect on *S. aureus* and *E. coli*.^92^ However, immobilization of silver sulfide (Ag_2_S) quantum dots with poly(amidoamine)-grafted MWCNTs has demonstrated microbial growth inhibition by 55.7% for *S. aureus*, 97.8% for *E. coli*, and 78.5% for *P. aeruginosa*. Besides, Ag_2_S-MWCNTs showed better antimicrobial activity as compared to cadmium sulfide quantum dots coated-MWCNTs.^73^

### MWCNTs blended with noble metals

For more promising results, MWCNTs were also blended with other noble metals, such as copper nanoparticles, to reduce the viability of bacteria by 75%.^93^ Likewise, bacteria (*E. coli*) treated with zinc oxide-coated-MWCNTs showed strong antimicrobial activity.^94^ A nanocomposite complex comprises of MWCNTs, titanium, and gold have demonstrated significant microbial growth inhibition against *B. subtilis*, *K. pneumoniae*, *S. aureus*, *C. albicans*, *Streptococcus pneumoniae*, *Proteus vulgaris*, and *Shigella dysenteriae*.^95^ Besides, titanium alloy-coated-MWCNTs blended with rifampicin were able to inhibit the formation of biofilm for up to 5 days.^27^

### Immobilized enzyme onto MWCNTs

Some enzymes like chloroperoxidase (CPO) and laccase were immobilized on the surface of MWCNTs to reduce the viability of bacteria by 99% for *S. aureus* and *E. coli*. The laccase immobilized with MWCNTs inhibited the microbial growth and spore formation for *B. cereus* and *B. anthracis* by > 99%.^96^ In order to understand their bactericidal mechanism, CPO catalyzed the oxidation of chloride into HOCl by H_2_O_2_ in the acidic conditions. The rapidly produced HOCl responds to H_2_O_2_ to provide singlet oxygen. Both singlet oxygen and HOCl are strong oxidants and have a wide range of antibacterial activity that may be exploited to inhibit, control, or reduce microbial growth. Moreover, the solution-phased CPO catalysis was very effective against *E. coli* and *S. aureus*.^96^ In contrast, the main antimicrobial agent in the laccase + methyl syringate (MS) system is the hydroxyl radical, which may be produced in different ways. For example, one of the mechanisms is called Haber–Weiss reaction that produces hydroxyl radical from H_2_O_2_ and superoxide radical. Once superoxide radical is produced by the one-electron MS radical transfer to O_2_, it may efficiently undergo dismutation to H_2_O_2_ that causes the destruction of bacterial cells.^96^

### Polymers adsorbed onto MWCNTs

The antimicrobial activity has also been observed for MWCNTs combined with different polymers. Murugan et al studied the antimicrobial activity of MWCNTs modified with dendrimer, such as amphiphilic poly(propyleneimine) and found that the microbial growth was inhibited by 87% for *E. coli*, 96.6% for *B. subtilis*, and 96.5% for *S. aureus*.^71^ The MWCNTs prepared by Neelgund et al with aromatic polyamide dendrimer exhibited a good antimicrobial effect on *P. aeruginosa* (65.2%) and *E. coli* (72.6%).^72^ In contrary, MWCNTs functionalized with poly(amidoamine) were consistently inhibited all selected bacterial growth.^73^ The antimicrobial activity of nanocomposites can be enhanced with increasing concentrations of MWCNTs. This is proven by Goodwin et al where MWCNT-poly (vinyl alcohol) has successfully reduced the viability of bacteria with increasing concentrations of nanotubes.^49^

### MWCNTs-based hydrogels nanocomposite

Currently, the antimicrobial activity of MWCNTs-based chitosan hydrogels has been extensively studied due to the physiological nature of the hydrogel-based materials. Interestingly, chitosan has also been previously used as an antimicrobial agent in many studies. For instance, a strong antimicrobial activity of MWCNTs-based chitosan hydrogels against *S. aureus*, *E. coli*, and *Candida tropicalis* was observed.^80^ Mohammad et al also reported that MWCNTs-based chitosan hydrogel exhibited a wide range of antimicrobial activity.^81^

## The antimicrobial mechanisms of CNTs

**Figure 1:**
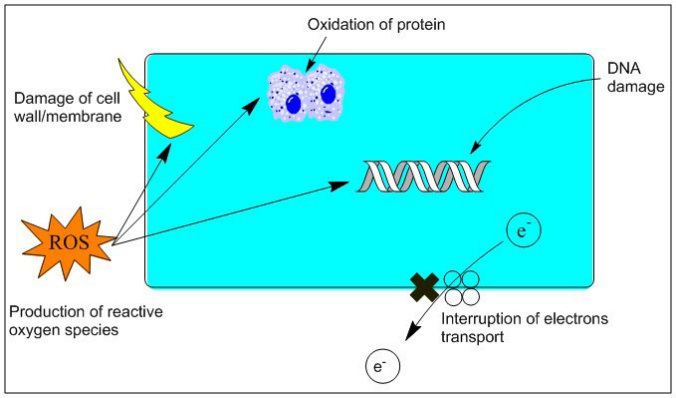
Antimicrobial mechanism of CNTs (Reprinted with permission from Li et al; Copyright, Elsevier).

Various antimicrobial mechanisms have been proposed in previous literature, with one of the examples shown in Figure 1: (1) attachment of CNTs on the microbial cell surface to promote the transmembrane electron transfer and induce cell wall and membrane damage; (2) protein dysfunction and DNA damage when CNTs penetrating bacterial cells; (3) formation of secondary products, such as ROS.^97^

Many studies reported that the destruction of pathogens cell membrane causes the leakage of intracellular contents and then followed by the death of microbial cell. For instance, Kang et al reported the first direct evidence that bacterial cell membrane damage occurred due to the direct contact between SWCNTs and pathogen, resulted in the leakage of intracellular contents such as DNA, RNA, and protein.^15^ Few studies observed that 60 minutes contact time between bacteria and SWCNTs was sufficient to destroy the membrane, whereas others demonstrated that a longer time (up to days) was needed to obtain the same results.^15-17,46,98^ Arias et al studied the physical contacts between microbial cells and aggregated SWCNTs using scanning electron microscopy (SEM) and reported the side walls of SWCNTs interacted with the *Salmonella* cells.^46^ The mechanism that describes the death of bacterial cells often starts from the destruction of cell membranes and then followed by the discharge of intracellular materials. The adherence of CNTs alters the cellular structure, permeability, and proton motive force of the cellular membrane. Some studies have observed that CNTs tend to distort the cell morphology and the cellular membrane integrity when brought to contact with bacterial cells. Kang et al studied the loss of bacterial (*E. coli*) cellular integrity by SEM and confirmed the cytoplasmic contents efflux by measuring the concentration of RNA and DNA.^17^ With the presence of SWCNTs, a two-fold increase of RNA and five-fold increase of plasmid DNA were found in the solutions, indicating the severe damage in cellular membrane integrity.^17^ Similarly, Saleemi et al reported the antimicrobial mechanisms of double-walled and multi-walled CNTs against *S. aureus*, *P. aeruginosa*, *K. pneumoniae*, and *C. albicans*.^21^ They found that both types of CNTs were wrapped around the surface of pathogens and caused severe damage to the cell wall/membrane of the selected pathogens.^21^ Furthermore, Liu et al suggested that the dispersed SWCNTs were acted as “nano darts” in the solution, attacking both Gram-negative and Gram-positive bacteria, which convincingly increased the bacterial cell death.^99^ Another similar result was reported after bacterial cells (*Ralstonia solanacearum*) were incubated with CNTs.^100^

### Generation of oxidative stress and ROS

Oxidative stress is considered as the main mechanism to induce the toxicity of CNTs in microbial cells. When CNTs penetrate the microbial cells, the ROS are generated, including hydrogen peroxide (H_2_O_2_), superoxide anion (O_2_•_), organic hydroperoxides, and hydroxyl radicals (OH•). The oxidative stress produces these free radicals and initiates the unsaturated phospholipids peroxidation in cellular membranes, which thereafter generating peroxyl radical intermediates that cause the severe destruction of nucleic acids and lipoproteins. The lipid peroxidation also induces membrane malfunction by changing the membrane fluidity. This has caused the conformational variations in membrane proteins, resulting in bacterial cell death.^101^

The physicochemical properties of CNTs, such as electrophilic nature and surface area, are the factors to determine the amount of production of ROS in bacterial cells. Bacterial cell destruction occurs when the activities of the antioxidant enzyme are damaged by excessive ROS generated inside the cell. After bacterial cells were exposed to CNTs, the genes (part of oxyR and soxRS systems) that are associated with oxidative stress were expressed.^16,17^ On the other hand, Vecitis et al investigated the toxicity mechanism of SWCNTs against *E. coli* using *in vitro* model of SWCNTs-mediated glutathione oxidation, a redox state mediator and non-enzymatic antioxidant that protects the pathogen from oxidative stress.^22^ Their results indicate a rise in glutathione oxidation and the lipid peroxidation in the microbial membrane with an increasing fraction of metallic SWCNTs. The imbalance of both oxidant and antioxidant therefore causes oxidative stress to increase inside the bacteria. In addition, it has been proved that toxicity of C_60_ is primarily caused by the oxidative stress on microbial cells. DNA microarray report showed that variations in the expression of oxidative stress-related genes were observed after the cells were treated with CNTs.^16^ However, some studies indicated that oxidative stress is not the only factor to cause microbial cell death. For instance, a study conducted by Liu et al assessed the oxidation-reduction capacity of SWCNTs and reported the loss of thiol groups (-SH) on the proteins both outside and inside the cell membranes of *Bacillus subtilis* and *E. coli* after treated with SWCNTs.^99^ Under anoxic conditions, no thiol oxidation was observed following treatment with SWCNTs, indicating that SWCNTs remained outside the microbial cell and were unable to penetrate the cell membrane to oxidize the intracellular proteins. These results suggested that oxidative stress produced by SWCNTs may not play a significant role in the antimicrobial activity.^99^

### Destruction of DNA

**Figure 2:**
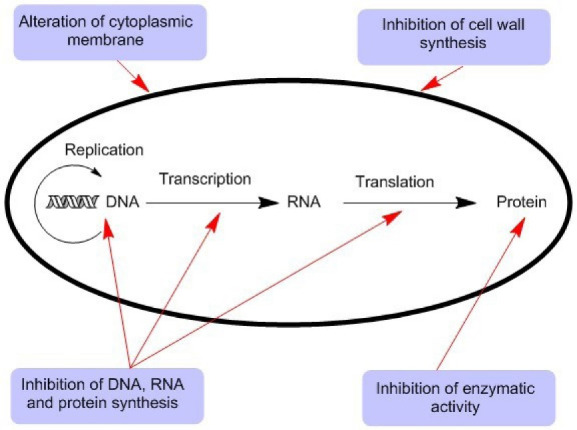
The different antimicrobial mechanisms of nanomaterials.

Many studies have reported that the adhesion of CNTs to microbial cells is the main trigger of the antimicrobial effect. However, CNTs may attach to the surface of bacteria (*S. mutans*) through entanglement without bacterial cell membrane damage, as reported.^102^ Simon-Deckers et al studied the adsorption of bacteria (*Cupriavidusmetallidurans* or *E. coli*) onto the CNTs (MWCNTs) by transmission electron microscopy and found that CNTs can induce protein dysfunction and DNA damage.^103^ The direct contact between CNTs and DNA could cause the destruction of DNA dominated through the single-strand breaks. Moreover, CNTs may diminish the power of supercoiled DNA base stacking and make the conformational variation in DNA. In general, nanomaterials can induce antimicrobial effects by destroying the cell membrane of bacteria or by passing through the membranes and specifically targeting the intracellular components such as protein, RNA, and DNA, as shown in Figure 2.^104,105^

Nonetheless, CNTs can also obliquely contact with DNA without entering the cell. This can be accomplished by secondary effects (such as ROS), with the free radicals generated through the interaction of CNTs with the cellular environment. For instance, the ROS may interact with the DNA, causing changes in the structure of DNA and thereafter inhibiting the repairing mechanisms.^106^ CNTs have the tendency to bind with the side chains of amino acid and SH groups of proteins to reduce their electrical properties.^107^ Besides, CNTs contain metallic catalytic residues (e.g. nickel) during their synthesis process, where this transition metal (nickel) is involved in the Fenton reaction to generate hydroxyl radicals that react with the protein molecules.

## Toxicity/biosafety profile of CNTs

CNTs have been extensively used in various biomedical applications. However, the detailed biosafety profile should be further investigated due to their toxicological effects on the human body. The high surface area to volume ratio of CNTs has increased their absorption rate, but this could possibly induce high toxicity and reactivity to the biological system and the environment, as reported.^108,109^ The authors proposed that the interaction of CNTs with the biological systems can induce cytotoxic effects, such as ROS production, allergy, DNA damage, cytotoxicity to normal cells, and protein dysfunction.^110,111^ The toxicity of CNTs depends on their size, shape, types of coating, aggregation, reactivity, mode of interaction with cells, and types of cell and tissue.^112-114^ Even though the research data on the toxicity assessment of CNTs through *in vivo* and *in vitro* study models is still limited, the toxicity profile of CNTs must initially be evaluated before widely used as microbial growth inhibition agents.

Despite the significant number of achievements that has been made in the research of CNTs-polymer nanocomposites over the last decade,^115-117^ there are some drawbacks to be taken into consideration. For example, weak interfacial bonding and poor dispersion remain a big problem for successfully integrating CNTs into the polymeric matrices. There are many challenges experienced when investigating CNTs as filler nanomaterials to be resolved. A homogeneous dispersion of CNTs in the polymeric matrix is difficult to accomplish and requires a strong interfacial interaction between polymeric matrix and nanotubes.^116,118^ Previously, researchers have tried to efficiently reinforce CNTs with the polymeric matrix.^119,120^ The homogeneous dispersion of CNTs is crucial to proficient reinforcement in the polymer nanocomposites.^121^ Much efforts have been made to improve the CNTs dispersion, such as physical treatment, and chemical- and surfactants-based modifications of CNT’s surface.^120,121^ Notably, a strong interfacial interaction is very important to take full benefit of the unique properties of CNTs. Therefore, functionalization of CNTs has been proposed to enhance the bonding at the interface and to significantly improve the dispersion of CNTs.^121,122^

## Application of CNTs in urinary tract devices

**Figure 3:**
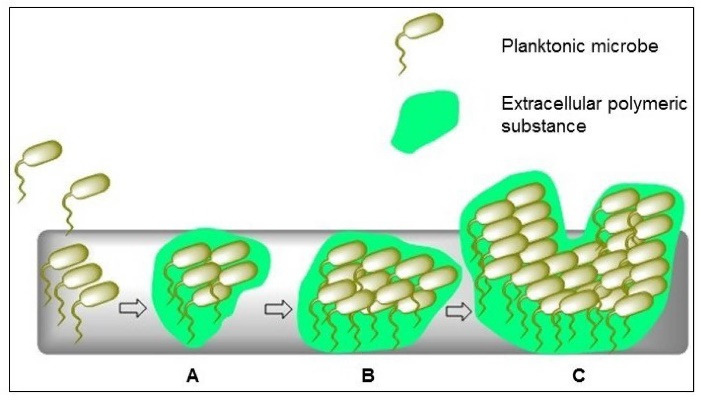
Schematic illustration of biofilm formation strategy. (A) Planktonic or free-floating bacteria attached to the surface. (B) Formation of bacterial self-produced extracellular polymeric substance (EPS) and colonized on the surface to produce a complex three-dimensional structure. (C) Bacterial communities produced within hours.

Due to the remarkable physicochemical properties of CNTs, there are numerous CNTs-based devices that have been extensively used in various biomedical application, such as urinary tract devices (e.g., the ureteral stents and urinary catheters) used in clinical practice even though it may cause urinary tract infections (UTIs) in some cases. Previous studies reported that UTIs were associated with 17% of hospital-acquired infections and had a prevalence rate of 27% and 36% in Europe and USA respectively.^123,124^ The formation of biofilm by microorganisms is adhered to the inert or living surfaces, then surrounded by self-produced extracellular polymeric substances, and promotes bacterial growth within hours (see Figure 3).^104,125^ Such process can cause significant harmful effect on human.^126^ Hospital-acquired infections are the major cause of mortality in the United States and the infection rate is 65% due to microbial biofilms.^127^ Most of the infections are initiated from medical instruments, such as bladder catheters (10~30%), fracture fixation and dental implantation devices (5~10%), and heart assistant instruments (25~50%).^128^

To overcome this drawback, application of various polymers has been implemented for biomedical instruments.^129^ For instance, silicone polydimethylsiloxane (PDMS) has been applied in the urinary catheters and several implants primarily for the vesicoureteral reflux correction in the bladder.^130,131^ The pathogen (*E. coli*) which is associated with 80% of UTIs may re-emerge and persist in the bladder after antibiotic treatment.^132-134^ Moreover, the cost of replacing the infected implants during revision surgery can be twice the cost of primary implant operation.^125^

On the other hand, the biofilm control approach has been suggested to reduce the infection, where the coating surfaces are used to discharge the antimicrobial agents over time. This approach comes with some limitations, including their toxicity to human cells, uncontrolled discharge of agent, and depletion of antimicrobial molecules, and the microbial resistance development.^26,135^ In addition, more promising approaches have been applied to control the biofilm formation, such as reducing initial cellular adhesion via the physical methods which are unlikely causing bacterial resistance.^29,136^ Thus, PDMS can be widely used in the membrane biofilm reactors for the treatment of wastewater due to its great cell adhesion properties, and to develop valuable compounds.^137-141^

Recently, the authors have successfully incorporated MWCNTs into the PDMS in order to control the adhesion of bacteria (*E. coli*).^142^ They found that small amounts of pristine MWCNTs (0.1%) caused a reduction by 20% on bacterial adhesion, while the oxidized form of MWCNTs (treated with nitric acid) also increased 20% of bacterial adhesion. These results matched by an earlier study conducted by Arias and Yang, where functionalized MWCNTs (with functional group (-OH)) did not show considerable antimicrobial activity against pathogens.^46^ In contrary, Chen et al have demonstrated that functionalized MWCNTs (with functional group (-OH) showed significant dose-dependent microbial growth inhibition.^19^ Therefore, both studies indicated that the surface performance may be affected by specific experimental conditions. Consequently, more studies need to be carried out to investigate further the behaviors of the MWCNTs/PDMS composites in microbial growth environment and inhibit the biofilm formation on the biomedical devices.^142^

**Figure 4:**
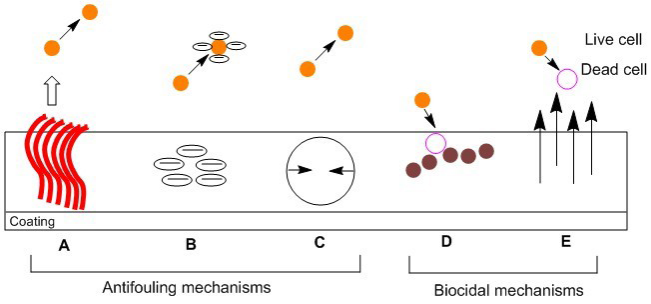
Antifouling and biocidal mechanisms of nanomaterials. (A) Steric repulsion: nanomaterials incorporated with the coating surfaces that provide physical berries to microbes, proteins, and cells. (B) Electrostatic repulsion: prevent the attachment of pathogens due to charges on the coating surfaces. (C) Low surface energy: reduction of external bacterial adhesion. (D) Releasing of biocide: start killing the microbes by the release of silver ions and nitric oxide. (E) Contact-active: kill multi-drug resistant (MDR) pathogens upon contact with the coatings.

However, CNT-polymer nanocomposites have been widely applied in the medical and pharmaceutical fields, often displaying significant developments in the thermal, mechanical, optical, and electrical properties of the nanocomposites as compared to polymer alone.^143^ In addition to the use in the fabrication of biosensors, CNTs have been applied in the development of drug delivery systems due to their immense potential in the biomedical field.^5,25,144^ Previous studies showed that CNTs can be adhered to the cell membrane and used as coatings for medical implants to enhance the cell growth and attachment.^145-147^ Similarly, incorporation of CNTs into polymers has demonstrated to increase the cell proliferation and attachment, with significant effects in tissue engineering scaffolds and cell culture substrate.^80,148-151^ In case of implantable medical devices, bacterial adhesion on the surface of implant often causes implant failure and severe infections. Therefore, the antifouling properties of CNTs have rendered them as potential nanomaterial for a broad range of biomedical applications.^24^ The antifouling coatings do not specifically kill the pathogen, but to prevent bacteria from being attached to the surfaces that allow biofilms to form.^152,153^ In general, the antifouling mechanisms of nanomaterials involve exclusion steric repulsion, low surface energy, electrostatic repulsion, releasing of biocide, and killing of microbes upon direct contact with the coatings, which inhibit the biofilm formation by plankton bacteria, as shown in Figure 4.^104^ The antibacterial activity of CNTs mainly depends on the number of graphene layers, aspect ratio, and physical disposition.^154^ Previous studies demonstrated the effectiveness of MWCNTs nanocomposites in the removal of bacterial adhesion and biofilm.^40,80^ For instance, PDMS is widely used in the production of implants and medical devices in the biomedical industry.^155^ Specifically, in the manufacturing of UTI devices, PDMS is usually applied due to its great biocompatibility, excellent chemical stability, and mechanical resistance.^156,157^ However, the use of PDMS in the biomedical sector carries some drawbacks, such as it is vulnerable to non-specific surface attachment of pathogens and protein. Currently, various studies reported the enhancement of antibiofouling characteristics of PDMS by the attachment of MWCNTs.^158,159^

With respect to PDMS/CNTs nanocomposites, various reports have been conducted on their thermal, electrical, and mechanical properties, suggesting that CNTs attachment may be beneficial.^160-163^ Another best example of these composites’ application is the production of electrically conductive materials for biomedical implants with sensing ability.^164,165^ Thus, a detailed analysis at the interface level could provide insights into the application of CNTs-based coatings in different medical implants.

## Conclusion and outlook

CNTs are remarkable nanomaterials for various biomedical applications, specifically used in developing the antimicrobial surfaces. The antimicrobial properties of CNTs depend on multiple factors, which respectively affect the overall performance. For instance, the surface functionalization of CNTs plays a crucial role to improve their biocompatibility and hydrophilicity. There are different other materials, such as metals, polymers, and biomolecules, which could be blended with nanotubes for developing effective CNTs-based nanocomposites, that show high antimicrobial activity towards a wide range of microorganisms. Some studies have proposed that SWCNTs are more effective in microbial growth inhibition than MWCNTs, while there are some other studies supporting the use of MWCNTs for antimicrobial activities. This proves that the antimicrobial mechanisms of different types of CNTs are yet to be fully understood. Consequently, further research work is required for the development of CNTs-based nanocomposites to produce new antimicrobial surfaces. The toxicity or biosafety profile of CNTs-based nanocomposites should also be carefully studied before they can be widely used in biomedical applications.

Carbon nanostructures (CNSs) emerged about three decades ago, with significant development progress been reported over a short period of time. In recent times, most of the carbon nanomaterials are still under extensive research to discover their potential as an antimicrobial agent. While many carbon-based products are commercially available, other antimicrobial materials such as AgNPs and polymers are still preferred for three main reasons; (1) large-scale production of CNSs is challenging, (2) the production of CNSs is costly and time consuming, and (3) the cytotoxicity/biosafety profile of CNSs has not been fully interpreted.^166^ Therefore, upcoming research should primarily focus on the large-scale production of non-toxic CNSs at minimal cost. Furthermore, the functionalization of CNTs seems to convincingly increase the overall efficiency in various biomedical applications, paving the way for broad integration in biomaterials. While safety profile of CNTs still needs to be carefully investigated, the production of new biomaterials for nanomedicine application will require their demand and superiority in the near future.

## Acknowledgments

This project was supported by the Research Collaboration between Faculty of Health and Medical Sciences, and Faculty of Innovation and Technology, Taylor’s University Lakeside Campus, Malaysia under the Flagship Grant with project code: TUFR/2017/001/05.

## Ethical Issues

Not applicable.

## Conflict of Interest

The authors report no conflicts of interest.
